# Models of Disability in Children’s Pretend Play: Measurement of Cognitive Representations and Affective Expression Using the Affect in Play Scale

**DOI:** 10.3389/fpsyg.2017.00794

**Published:** 2017-05-18

**Authors:** Stefano Federici, Fabio Meloni, Antonio Catarinella, Claudia Mazzeschi

**Affiliations:** Department of Philosophy, Social and Human Sciences and Education, University of PerugiaPerugia, Italy

**Keywords:** models of disability, play of children, pretend play, affect in play scale, medical model, social model, ICF, causal origins of disability

## Abstract

Play is a natural mode of children’s expression and constitutes a fundamental aspect of their life. Cognitive, affective, and social aspects can be assessed through play, considered as a “window” to observe a child’s functioning. According to Russ’s model, cognitive and affective components and their reciprocal connections can be assessed through the Affect in Play Scale (APS). The aim of the present study was to investigate children’s representations of the three main models of disability (medical, social, and biopsychosocial) and how these models affected cognitive and affective components of children’s play. Sixty-three children, aged 6–10 years, were assessed by means of the APS. Participants were randomly assigned to one of two APS task orders: the standard APS task followed by the modified APS task (including a wheelchair toy), or vice versa. The standard and modified APS sessions were coded according to the APS system. The modified APS sessions were also coded for the model of disability expressed by children. A one-way ANOVA conducted on the APS affective and cognitive indexes revealed an effect of condition on the affective components of play and no effect on cognitive components and variety of affect as assessed by the APS. In addition, when children are involved in pretend play from which concepts of disability emerge, these concepts are almost exclusively related to the medical model of disability. Results suggested implications for intervention with children in educational contexts that aim to teach children about disability.

## Introduction

Play is a natural mode of expression of children and constitutes a fundamental aspect of children’s life (Nicolopoulou, 1993). Play can be considered a window through which one can observe a child’s cognitive, affective, and social functioning (Stagnitti, 2004). In particular, pretend play is a complex behavior involving symbolic expression of thoughts and feelings (Cherney et al., 2003; Russ, 2004). Recent evidence indicates that pretend play has important connections to the development of cognitive, affective, and social skills (Pellegrini and Smith, 2005). These connections make pretend play an important phenomenon to study, due to its capacity to provide insight into children’s abilities and internal representations (Niec and Russ, 2002) and its appropriateness for investigating the “architecture” of the child’s mind (Weisberg, 2015).

In order to grasp the complexity of play, it seems necessary to evaluate it in terms of the dimensions of pleasure expressed, contents/themes, and linguistic and structural aspects, through a meaningful theoretical and methodological model founded on evidence-based procedures (Lewis J. M., 1993; Mash and Hunsley, 2005).

Russ developed a model of pretend play and a scale to measure the play of children from 6 to 10 years old, the Affect in Play Scale (APS) (Russ, 1993, 2004). The APS qualitatively and quantitatively assesses affective and cognitive components of symbolic play, through the use of a standardized coding system that allows for measuring affective dimensions in fantasy and cognitive dimensions of play.

In the present study, Russ’s (2004, 2014) model, and its related APS, were used to evaluate the implicit component of the child’s internal representations of disability in the context of symbolic play. The APS had never before been used to evaluate fantasy and expression of affect in symbolic play involving characters with a disability. A modified version of the APS play task was used by introducing a toy wheelchair among the standard play task materials which could be worn by a puppet to represent a character with a disability.

To date, the scientific literature has identified three main models of disability: individual (including the medical model), social, and biopsychosocial (Bickenbach et al., 1999; World Health Organization [WHO], 2001). The individual model posits that disability is a direct consequence of disease, trauma, or other health conditions and, hence, that disability is an individual problem. In other words, it represents an individual-level deviation from biomedical norms of structure or function that requires medical care and treatment from health professionals (Boorse, 1975, 1977; Bickenbach et al., 1999; Pompili et al., 2013). Since treatment by the medical profession is central to this model, it is also referred to as the medical model of disability (Finkelstein, 1980; Oliver, 1981, 1996; World Health Organization [WHO], 2001). Unlike the individual model, the social model portrays disability as a cultural construct, the product of a particular social environment (Oliver, 1990; Roulstone et al., 2012). Physical barriers or social attitudes are considered the origin of disability, because they prevent individuals with a “disability” from gaining access to virtual and real spaces and make social participation difficult. In this model disability is not an individual attribute, but rather a complex collection of conditions which require social actions rather than just medical treatment (World Health Organization [WHO], 2001). Finally, since 2001 the *ICF: International Classification of Functioning, Disability and Health* has used a biopsychosocial—also known as interactive (Bickenbach, 2012)—model of human functioning and health, which represents an attempt to integrate the conflicting medical and social models. To achieve this “synthesis” (World Health Organization [WHO], 2001), disability is treated not as a consequence of disease but as the outcome of three variables related to human health: health status, environment, and personal factors. In **Table 1**, the main concepts of the three models of disability were summarized.

**Table 1:** Summary of the concepts of the tree main models of disability.

Model of disability	Causes of disablement	The model’s focus	Interventions
Medical/Individual	Consequences of disease or disorder	Sick individual who requires medical treatments provided by health professionals	Rehabilitative to “fix” the sick individual
Social	Social barriers	Cultural stereotypes, medical norms, and environmental hindrances	Political and cultural to break down social barriers
Biopsychosocial	Components of health (health structures and functions, personal and environmental factors)	Human beings functioning in their context	Shaping the health system to meet the individual’s functioning needs, and minimizing social barriers

Disability is a complex phenomenon and models of disability allow people to make sense of emotional responses to disability, to process thoughts and structure knowledge about disability, and to make decisions and judgments to which it is relevant (Brewer, 1999). Children’s models of disability were first studied by Meloni et al. (2015), who reported that the youngest group of children (6–8 years old) thought of people with disabilities as being sick. This early representation of disability is consistent with the individual model of disability and was independent from parents’ explanations and representations of disability. Older children (9–11 years old) had more knowledge of disability and endorsement of stereotypical beliefs tended to be lower, as children tended to espouse their parents’ representations.

Research based on Piagetian stage theory (Piaget, 1929, 1952, 1954) claimed that young children’s difficulty in conceptualizing disability was the result of their being too cognitively immature (Lewis A., 1993; Lewis, 1995; Glasberg, 2000). According to these approaches, children can only process and structure knowledge about disability across a range of explanations for disabilities identifiable among the three main models of disability, which include physical, biological, and psychological causes of disablement and health, when they reach approximately 11 years of age. Although debates continue, studies of cognition in infancy demonstrate that knowledge begins to emerge early in life and constitutes part of humans’ innate endowment (Baillargeon et al., 1985; Spelke, 1994; Baillargeon et al., 1995), even including an early understanding of disease causality (Springer and Ruckel, 1992; Sigelman et al., 1993). In line with this work, challenging Piagetian framed research on children’s knowledge of illness causation, Smith and Williams (2004) explored children’s understanding of the causal origins of disabilities. They found that children of all ages showed a preference for physical and biological causes of disability (consistent with an individual/medical model) and rejected social–psychological causal explanations (consistent with a social model).

The aim of the present paper was to explore the type of representation of disability and the effect of the presence of a disabled character on the expression of affect and on the cognitive dimensions of children’s play. In accordance with previous findings (Smith and Williams, 2004; Meloni et al., 2015), we expected that, when one of the two puppets in the modified APS play task was in a wheelchair (disabled puppet), children would tell stories in which the psychological constructs, sequence of actions, and affective expressions were consistent with the individual/medical model of disability. We also expected that pretend play with a disabled puppet would display a strong connection between affective categories such as nurturance/affective and sadness/hurt and actions such as providing and receiving care.

## Materials and Methods

### Participants

Thirty Italian 6- to 10-year-olds were recruited from a public primary school. All of them were Caucasian, attending mainstream classes and had no declared disability, and sixteen (53.3%) were female. Parents provided written, informed consent to their children’s participation in the study and to the videotaping of their children in the play session. All the children also consented personally after the researcher explained that he or she would like to watch the child playing with two puppets for a few minutes. The Ethics Committee of the Department of Philosophy, Social and Human Sciences and Education at the University of Perugia reviewed and approved the study. The study presented “no more than minimal risk”.

### Instrument

*The standard APS* (Russ, 2004) is a standardized instrument for evaluating cognitive and affective dimensions in pretend play in children from 6 to 10 years of age, based on an observational procedure that focuses on different children’s behaviors during a semi-structured, 5-min, evidence-based play task. The APS has been employed in numerous studies that have demonstrated its good psychometric characteristics. Good inter-rater reliability was achieved, with Cohen’s kappa values ranging from 0.70 to 0.90 (Russ, 2004). The APS play task is video recorded and requires two neutral-looking hand-puppets, representing a boy and a girl, and some little wooden blocks. The instructions are standardized and facilitate the child playing freely, according to his or her skills, age, characteristics, and preferences. The researcher introduces the two puppets and the blocks to the child and asks him or her to play with them for five minutes.

*The modified APS*, an adapted APS play task procedure, was developed for this study and involved changes to the APS materials and instructions. The modified APS play task included a wheelchair toy in addition to the two puppets and the blocks. The experimenter introduced the wheelchair toy (wearable by one puppet) and asked the child which of the two puppets (boy or girl) was disabled and which was not.

Henceforth, the terms “standard APS” and “modified APS” are used to refer, respectively, to the original instrument (Russ, 2004) and to the version developed for this study.

*A semantic discrimination task* (Meloni et al., 2015) was administered to assess children’s comprehension of the concept “disabled.” The child was presented with six stimuli (2 photographs of people with disabilities; 2 photographs of people without disabilities; 2 words: “handicapped” and “normal”) and asked sort them by placing them in one of two labeled baskets. The baskets were labeled “disabled” and “normal.” A child was considered to have passed the test if he or she demonstrated understanding of the difference between the semantic categories represented by the stimuli. The test was repeated until the child had either demonstrated that he or she could correctly discriminate between the stimuli or it was clear that he or she was unable to do so.

### Measures

#### The APS Rating Scale

The APS rating scale (Russ, 2004) was used to analyze the standard and the modified APS play tasks. The APS scores used in the present study belong to two domains: affective and cognitive.

Affective domain:

(1) *Total Frequency of Affective Expressions score*: is measured by the sum of eleven affective categories (happiness/pleasure, nurturance/affection, oral, sexual, competition, anxiety/fear, sadness/hurt, frustration/disappointment, aggression, anal, and oral aggression). The categories can be applied to verbal or nonverbal expressions, and can be an affect state (“This is fun”) or an affect theme (“This bomb is going to explode”).
(2) *Frequency of Positive Affect score*: sum of the five affect categories: happiness/pleasure, nurturance/affection, competition, oral, and sexual.
(3) *Frequency of Negative Affect score*: sum of the six affect categories (aggression, sadness/hurt, anxiety/fear, frustration/disappointment, oral aggression, and anal).
(4) *Variety of Total Affect Categories score*: is a count of affect expressions across the 11 possible categories.
(5) *Variety of Positive Affect Categories*: is a count of affect expressions across the five positive categories.
(6) *Variety of Negative Affect Categories*: is a count of affect expressions across the six negative categories.

Cognitive domain, rated on a five-point Likert-type scale:

(1) *Organization*: includes the quality and the complexity of the play plot.
(2) *Elaboration*: measures the amount of embellishment in the play in terms of theme, facial expression, voice tones and character development.
(3) *Imagination*: involves number of ideas, novelty, and fantasy of the play in terms of the presence of themes outside of everyday experience.
(4) *Comfort*: rates the child’s overall level of enjoyment engaging in pretend play and her ability to be involved in play.

#### Representation of Disability

Representation of disability was only scored for the modified APS. Expressions were classified assigning them to one of three categories of disability model (medical, social, and biopsychosocial), as follows.

##### (i) Individual/Medical Model

Statements in which the disability was related to the health of the disabled puppet. This category also included statements on impairments assigned to the disabled puppet. This category includes: (i) all statements implying that the disabled puppet (i.e., puppet in the wheelchair) was considered morally or ethically responsible for his or her disability; (ii) any judgment based on the appearance of the disabled puppet, e.g., beauty, or ugliness; (iii) any statement assigning responsibility for the disability to an external spiritual, vital, or religious force.

##### (ii) Social Model

All statements that attributed the disability to factors beyond control of the disabled puppet, such as architectural and cultural environmental factors (barriers, rules, regulations, etc.), or to human attitudes and prejudices.

##### (iii) Biopsychosocial Model

As the biopsychosocial model is a composite, we included in this category articulations attributing disability to a complex interaction of medical, environmental, and socio-relational factors, including a clear reference to individual functioning (health or disease).

### Procedure

#### Administration Procedure

After a parent had provided written, informed consent for his or her child’s participation, the researcher explained to the child that he or she would like to learn about play by watching the child play with the two puppets for a few minutes and asked for the child’s own consent to this. All children were assessed individually.

First, the semantic discrimination task was administered to assess comprehension of the concept “disabled.” Then, children were invited to play using both the standard APS and the modified APS play task sequentially. The two sessions (standard and modified) were administered consecutively and were videotaped. The procedure lasted roughly fifteen minutes (5′ semantic discrimination task; 5′ standard APS; 5′ modified APS).

#### Coding Procedures

Recordings of the two play sessions (standard and modified) were transcribed verbatim and the video recordings scored, using the APS scoring system procedure (Russ, 2004), by two independent trained coders. The modified APS play task verbatim transcriptions were also scored by two independent trained coders. The score for each disability model (individual/medical, social, biopsychosocial) is obtained by summing the child’s expressions attributable to each model. Inter-rater reliability was assessed using the Pearson correlation coefficient on 20 randomly selected protocols. The correlations between the two judges for all the scores ranged from 0.87 to 0.94.

### Data Analysis

Descriptive statistics (mean, *M*; standard deviation, *SD*) were calculated to provide a profile of the sample. Inferential statistics (multivariate ANOVA and univariate ANOVA) were used to compare children’s play performance on the standard APS and modified APS, and *t*-tests for unpaired samples and effect sizes for Student’s *t-*test (Cohen’s *d*) were calculated to compare children’s play performance on the standard APS with normative Italian data. Chi-square tests were used to explore the association between children’s gender and models of disability, and correlational analysis was used to explore the association between children’s age and models of disability. Data were analyzed using IBM^®^SPSS Statistics 23.

## Results

### Sample

Fifty-five out of 63 primary school pupils invited to play completed the experiment (male: *n* = 28, 50.9%; female: *n* = 27, 49.1%; *M* age = 8.10 years, *SD* = 1.45, range: 6–10) (**Table 2**). Eight pupils stopped playing during one or both of the 5-min task periods, not playing after a 2-min period. For this reason, the people pupils were excluded from the analyses.

**Table 2:** Sample profile: school grade and gender.

	Male		Female		Total	
Grade	*N*	%	*N*	%	*N*	%
I	4	14.3	6	22.2	11	18.2
II	5	17.9	7	25.9	12	21.8
III	5	17.9	5	18.5	10	18.2
IV	5	17.9	4	14.8	9	16.4
V	9	32.1	5	18.5	14	25.5

**Total**	**28**	**100**	**27**	**100**	**55**	**100**

Twenty-five pupils played with the standard APS play task first.

### Affective and Cognitive Components in the Standard APS Play Task

Results for the APS standard condition are reported in **Table 3**. Values available from the normative Italian sample are reported in parentheses (Mazzeschi et al., 2016).

**Table 3:** Means and standard deviations for the sample.

	Frequency		Variability	
	*M*	*SD*	*M*	*SD*
Total Frequency of Affective Expressions	14.83 (12.61)	12.99 (11.79)	3.38 (3.38)	2.64 (2.21)
Frequency of Positive Affect	10.73 (7.63)	9.98 (8.53)	1.88 (1.74)	1.33 (1.28)
Frequency of negative Affect	4.11 (4.96)	4.78 (6.31)	1.58 (1.64)	1.67 (1.32)
Aggression	0.55 (1.50)	1.24 (3.71)	0.22	0.42
Nurturance/affection	1.35 (1.14)	2.06 (1.81)	0.42	0.49
Happiness/pleasure	6.50 (4.12)	5.36 (5.17)	0.75	0.44
Anxiety/fear	0.63 (0.76)	1.16 (1.87)	0.29	0.46
Sadness/hurt	0.78 (1.05)	1.62 (2.24)	0.33	0.47
Frustration/disappointment	1.22 (1.34)	2.27 (2.09)	0.39	0.49
Competition	0.36 (0.68)	1.42 (2.29)	0.09	0.29
Oral	2.11 (1.45)	4.53 (3.44)	0.36	0.48
Oral aggression	0.25 (0.08)	0.93 (0.47)	0.09	0.29
Anal	0.51 (0.25)	1.17 (0.91)	0.22	0.42
Sexual	0.36 (0.25)	1.11 (1.13)	0.13	0.34
Organization	2.42 (2.32)	1.28 (1.15)	–	–
Elaboration	2.36 (2.21)	1.27 (1.03)	–	–
Imagination	2.35 (2.21)	1.04 (1.03)	–	–
Comfort	2.73 (2.96)	1.31 (1.05)	–	–

*In parenthesis are data from the normative Italian sample.*

In order to compare children’s play performance on the standard APS with normative Italian data, *t*-tests for unpaired samples and effect sizes in terms of Cohen’s *d* were calculated. According to Cohen (1988), effect size values of 0.2, 0.5, and 0.8 are considered small, medium, and large. The comparison with data from the Italian normative sample indicated that, for the standard APS play session, children in the present sample displayed typical play, similar to the normative samples in all of the APS scores. The only two exceptions to this trend were the Frequency of Positive Affect (*t* = 2.61, *df* = 1264, *p* < 0.01, *d* = 0.333) and the happiness/pleasure category (*t* = 3.33, *df* = 1264, *p* < 0.01, *d* = 0.452). Both Cohen’s *d* results were of a medium size.

### Affective and Cognitive Components in the Modified APS Play Task

A one-way ANOVA of the frequency of the 11 affect categories revealed effects of APS condition on one category of positive affect, “nurturance/affection” [*F*(1,110) = 11.98, *p* = 0.01, η^2^ = 0.100], and one category of negative affect, “sadness/hurt” [*F*(1, 110) = 9.82, *p* < 0.01, η^2^ = 0.083]. An effect size measured using partial η2 of 0.01 constitutes a small effect, 0.06 a medium effect, and 0.14 a large effect. Both affect categories were more frequent in the modified APS condition (nurturance/affection: *M* = 3.93, *SD* = 5.13; sadness/hurt: *M* = 2.49, *SD* = 3.71). There was no effect of APS condition on cognitive components (Organization, Elaboration, Imagination and Comfort) and on variety of affect.

### Children’s Models of Disability

During the modified APS session, 29 out of 55 pupils expressed concepts relevant to a disability model (i.e., occurrence of a disability model). Twenty-six out of those 29 pupils only represented disability through the medical model, two through the medical and social models, and one through only the social model. None of the pupils used the biopsychosocial model. Amongst the 29 pupils who referred to disability in the modified APS session, the mean frequency of statements related to the medical model was 2.07 (*SD* = 1.28), while the mean frequency of mentions of the social model was 0.27 (*SD* = 0.92). Chi-square tests indicated that there was no relationship between gender and mentions of a disability model [χ^2^(1, *N* = 29) = 0.31, *p* = 0.58] and no relationship between gender and the relative frequency of the various disability models [χ^2^(5, *N* = 29) = 5.11, *p* = 0.40].

There was a correlation between age and mentioning at least one model of disability [*r*(55) = 0.27, *p* < 0.05]. There was no relationship between puppet gender and child’s assignment of puppet to the wheelchair in the modified APS.

Finally, **Table 4** shows which puppets were worn by children before starting timing of the two APS sessions (the child must put on the puppets before timing the session; Russ, 2004). Only one child wore the puppet with the wheelchair: 54 children never wore the puppet with the wheelchair freely (i.e., before the experimenter invited him or her to put on) (**Table 4**).

**Table 4:** Puppet wearing in the standard and modified APS play tasks.

Puppet(s) worn	Standard APS	Modified APS
Neither	16	20
Both	37	1
One (not-disabled)	2 (Males)	34 (male *n* = 15; female *n* = 18)
One (disabled)	–	0

A one-way ANOVAs on puppet wearing (1 = wearing neither; 2 = wearing both; 3 = wearing only one (non-disabled)) revealed an effect of APS condition (standard APS; modified APS) on puppet wearing, *F*(1,110) = 11.86, *p* = 0.01.

## Discussion

In line with previous studies (Nichols and Stich, 2000; Singer, 2002), the present paper claims that pretend play is a fundamental tool for investigating children’s affective, cognitive, and social functioning. Studying children’s pretend play can also provide insight into human cognitive architecture and its development (Weisberg, 2015).

With regard to the affective and cognitive components, children showed a play profile in line with Italian norms, playing in a typical way for their age (Mazzeschi et al., 2016). Children showed more expressions of empathy or sympathy and helping or supporting with another character (nurturance/affection) and more expressions of pain, sadness, or loneliness (sadness/hurt) when playing with the wheelchair toy (modified APS play task).

The organization and elaboration of the play plot and the child’s imagination and comfort in play, namely the cognitive components of the APS (Chessa et al., 2011), remained quantitatively unchanged across the experimental conditions (standard and modified APS play tasks). Disability—suggested by the introduction of the wheelchair toy in the modified APS—did not affect the construction of the structural components of the play plot and children remained available to symbolically play as usual. At the same time, children were shown to be very sensitive to the presence of the wheelchair toy, showing a general attitude of compassion and sadness toward disability. We infer that, from the point of view of the play plot, a wheelchair toy has the same weight as all the other play elements (hand-puppets and wooden blocks), by demonstrating that for a child one toy is like another. On the other hand, the child proves to know the emotional value of the wheelchair, since it is immediately associated with the disease and, therefore, with feelings of care, compassion, and sadness; this explains the variations measured in the affective components of play, but not the cognitive ones.

With regard to the children’s models of disability, data confirm the finding of Meloni et al. (2015) that, for children, individuals with disabilities are mainly thought of as being sick. When children are involved in pretend play, from which concepts of disability emerge, these concepts are almost exclusively related to the individual/medical model of disability. As we expected, the children imagine the disabled puppet as sick (e.g., disabled puppet says: “I’m sick because my legs are broken”), or requiring medical treatment (e.g., disabled puppet says: “The physician told me that within few days I’ll be better”), and the non-disabled puppet as a provider of health care (e.g., non-disabled puppet says to disabled one: “The physician told that you must take these pills that make you feel better”). These findings were reflected in the higher frequency of nurturance/affection and sadness/hurt in the modified APS condition, as expected.

As a cognitive organizer, a model of disability helps people to identify and understand the causal origins of disability (Meloni et al., 2015). The individual/medical model directs understanding of disability to the physical and biological condition of an individual, rejecting social and cultural determinants of disability. Therefore, our findings are in line with those of Smith and Williams (2004), who found that 4- to 11-year-old children showed a preference for physical and biological causes of disability and rejected social–psychological causal explanations.

The 26 pupils out of 55 who did not express concepts referable to a disability model were the youngest. It suggests that the capacity to tell stories in which disability is salient develops with age. At an early age, disability does not seem to attract children’s attention and is not featured in their stories. When disability is mentioned in a story, however, it emerges as the most salient element and drives the narrative. The disability element in children’s stories tends to conform mainly, if not exclusively, to ‘schemata’ (Brewer, 1999) from the individual/medical model of disability.

That the youngest did not express concepts referable to a disability model highlights what Smith and Williams (2004) suggested with regard to open-ended verbal methods: young children may have been so concerned with spontaneously generating a causal explanation that they were unable to verbalize a cause. In fact, when a forced-choice paradigm is adopted, as in Smith and Williams (2004) and Meloni et al. (2015), young children show some causal knowledge of disabilities.

Another interesting finding related to puppet wearing behavior before starting timing of the two APS sessions. In the standard APS, the majority of children wore both puppets, whereas in the modified APS most children only wore the non-disabled puppet freely. This behavior seems consistent with studies by Park et al. (2003) and Meloni et al. (2012), which demonstrated that disability elicits disgust and, hence, avoidance behavior. As the medical model of disability schematizes an aspect of human diversity by providing a cognitive organizer, such as a “frame” (Minsky, 1975), the avoidance behavior provides a “script” (Schank and Abelson, 1977) as well. No wonder, then, that, despite voicing caring attitudes toward the puppet in the wheelchair, almost none of the children elected to wear this puppet freely.

## Conclusion

Findings from this study support the validity of the APS to evaluate differences in children’s play in different situations, confirming the validity of the scale in showing the affective nuances of the play of school-aged children.

Use of the APS also allowed for confirmation of what previous research on disability representations and attitudes in children aged 6–10 years suggested. In particular, the perceptual salience of disability increases as the age of children grows. In the present study, all children who paid attention to disability tended to only describe and explain it in its biological and physical dimensions, neglecting any social and cultural determinants of disability. Therefore, the perspective of disability emerging from the children’s attitudes in the APS play task was fully compatible with the individual/medical model of disability as defined in the scientific literature. This compatibility was further confirmed by the fact that sadness/hurt affect categories prevailed in children’s storytelling when they interacted with the disabled character (puppet in the wheelchair), along with the nurturance/affection category. In fact, the prevalence of sadness/hurt feelings is consistent with the personal tragedy view (Swain and French, 2000) on which the medical model of disability is grounded.

The results obtained also suggest that disability is strongly and stereotypically associated with a negative and unpleasant dimension of existence, providing evidence for a cognitive mechanism underpinning the cultural construction of the individual/medical model of disability. Moreover, according to Smith and Williams (2004) research on children’s understanding of the causal origin of disability, our results challenge Piagetian’s assumption (Piaget, 1929, 1952, 1954) that young children conceptualize disability with difficulty. Children, indeed, were not surprised by diversity of disability, and demonstrated possession of cognitive schemata to elaborate on it and congruent emotions to respond to it. On the basis of these results, we claim that the child’s education about disability should not have the aim to introduce the concept of diversity, but rather to enhance their views on disability, modeling the social and cultural dimensions of diversity among the disabled that seem to be lacking in children.

Future research might overcome some limitations of the present study. These include, for example, increasing the sample size, given that the sample of children recruited in the present study prevents us from generalizing the results as representative of the Italian child population. In addition, in order to better investigate the evolution of the concept of disability, a longitudinal study would be advisable. Finally, the present research reflects the Italian socio-cultural context, where the pressure to include disabled people in social life is a political and legal norm but not yet widespread in the social fabric. It would be interesting to see how the disability models feed children’s narratives on disability in other socio-cultural contexts.

## Author Contributions

Conceived and designed the experiments: SF and FM. Performed the experiments: AC. Analyzed the data: SF, FM, AC, and CM. Contributed analysis tools: SF, FM, AC, and CM. Wrote the paper: SF, FM, AC, and CM.

## Conflict of Interest Statement

The authors declare that the research was conducted in the absence of any commercial or financial relationships that could be construed as a potential conflict of interest.
